# The Number and Transmission of [*PSI* ^+^] Prion Seeds (Propagons) in the Yeast *Saccharomyces cerevisiae*

**DOI:** 10.1371/journal.pone.0004670

**Published:** 2009-03-05

**Authors:** Lee J. Byrne, Diana J. Cole, Brian S. Cox, Martin S. Ridout, Byron J. T. Morgan, Mick F. Tuite

**Affiliations:** 1 Protein Science Group, Department of Biosciences, University of Kent, Canterbury, United Kingdom; 2 Institute of Mathematics, Statistics and Actuarial Science, University of Kent, Canterbury, United Kingdom; Universidade Federal do Rio de Janeiro (UFRJ), Instituto de Biofísica da UFRJ, Brazil

## Abstract

**Background:**

Yeast (*Saccharomyces cerevisiae*) prions are efficiently propagated and the on-going generation and transmission of prion seeds (propagons) to daughter cells during cell division ensures a high degree of mitotic stability. The reversible inhibition of the molecular chaperone Hsp104p by guanidine hydrochloride (GdnHCl) results in cell division-dependent elimination of yeast prions due to a block in propagon generation and the subsequent dilution out of propagons by cell division.

**Principal Findings:**

Analysing the kinetics of the GdnHCl-induced elimination of the yeast [*PSI^+^*] prion has allowed us to develop novel statistical models that aid our understanding of prion propagation in yeast cells. Here we describe the application of a new stochastic model that allows us to estimate more accurately the mean number of propagons in a [*PSI^+^*] cell. To achieve this accuracy we also experimentally determine key cell reproduction parameters and show that the presence of the [*PSI^+^*] prion has no impact on these key processes. Additionally, we experimentally determine the proportion of propagons transmitted to a daughter cell and show this reflects the relative cell volume of mother and daughter cells at cell division.

**Conclusions:**

While propagon generation is an ATP-driven process, the partition of propagons to daughter cells occurs by passive transfer via the distribution of cytoplasm. Furthermore, our new estimates of n_0_, the number of propagons per cell (500–1000), are some five times higher than our previous estimates and this has important implications for our understanding of the inheritance of the [*PSI* ^+^] and the spontaneous formation of prion-free cells.

## Introduction

The term ‘prion’ was coined to describe an abnormal protein conformer that promoted its normal native counterpart protein to re-fold to the abnormal conformation [1]. In addition to the association of prions with the transmissible spongiform encephalopathies (TSEs), at least five prions have also been identified in fungi [2]. These include the [*PSI* ^+^] prion that was first identified in the budding yeast *Saccharomyces cerevisiae* as a novel cytoplasmic genetic determinant [3] and that was subsequently shown to be the prion form of the translation termination factor eRF3 (Sup35p) which is encoded by the *SUP35* gene [4].

The cellular role of Sup35p in translation termination can be exploited in a phenotypic assay to determine the [*PSI*] status of any strain of yeast carrying either the *ade1-14* or *ade2-1* mutations. Normally these nonsense alleles lead to the accumulation of a red colony pigment and adenine auxotrophy (Ade^−^). However, when the [*PSI* ^+^] prion is present in the cell these nonsense alleles are suppressed to give a white Ade^+^ phenotype. The availability of this, and a range of other cellular and biochemical assays to monitor the behaviour of Sup35p and [*PSI* ^+^] *in vivo* [5], means that the [*PSI* ^+^] prion provides an excellent model system in which to study prion biology.

The [*PSI* ^+^] prion shows a remarkably high degree of mitotic stability, with prion-free cells emerging under normal laboratory conditions at a rate of ≤10^−6^ per cell division. We refer to the infectious proteinaceous agents that need to be generated and inherited to propagate the prion state, as propagons [6] i.e. prion ‘seeds’. Precise details of the molecular composition of these self-replicating hereditary particles are lacking, but they are likely to be conformationally distinct oligomeric forms of the underlying prion protein rather than the large amyloid-like fibres associated with prions (e.g. [7], [8] and similar conclusions are emerging from studies with mammalian PrP [9].

An important chemical tool for studying yeast prion propagation is guanidine hydrochloride, a reversible inhibitor of the molecular chaperone Hsp104 [10]–[13]. Hsp104 is a cellular factor essential for the continued propagation of all known native yeast prions [14]–[16]. The currently accepted model for the role of Hsp104 is that it generates new propagons by cleaving high molecular weight aggregates of the prion protein into smaller heritable oligomers i.e. propagons [17], [18], thereby allowing their numbers to keep pace with cell division. The addition of GdnHCl prevents the Hsp104p-mediated generation of new propagons and this leads to the dilution out of the remaining propagons in dividing cells. Eventually cells appear in the population that lack the propagons necessary to propagate the prion state and they become [*psi* ^−^].

The process of elimination of [*PSI* ^+^] cells from a population of cells is referred to as ‘curing’. Curing of the [*PSI* ^+^] prion from growing yeast cells by the addition of 3 to 5 mM GdnHCl typically occurs over a 30–32 hr (i.e. 12–16 generations) time period. Initially a lag phase of 10–12 hr (i.e. 4–6 generations) is observed before [*psi* ^−^] cells begin to appear in the population [19], [20]. The length of this lag phase corresponds to the length of time it takes for the number of propagons to decrease to such small numbers that upon subsequent division, a daughter cell will fail to receive any of the remaining propagons prior to cytokinesis.

Data collected on numbers of [*PSI* ^+^] and [*psi* ^−^] cells over time in a GdnHCl-treated culture (i.e. curing data) can be modelled mathematically to allow the estimation of the mean number of propagons present in a [*PSI* ^+^] cell prior to the addition of GdnHCl [19], [21], [22]. Our original model has undergone several iterations, with the aim of better reflecting the biological processes that impact on prion propagation and transmission. The most sophisticated of these models [22] is a multitype branching process that allows one to estimate the mean number of propagons in a [*PSI* ^+^] strain and the proportion of propagons transmitted to a daughter cell following cell division in the presence of GdnHCl, using the method of maximum likelihood. However, to fit this model to curing data certain information about cell reproduction is also required. This information comes from estimates of the population growth rate parameter (or Malthusian parameter [23]) and from detailed cell reproduction experiments following individual cells under a microscope, which can be used to estimate cell lifetime distribution parameters.

Varying the growth rate of mother and daughter cells, and altering the propagon distribution between the two can dramatically affect the estimate of *n*_0_ and therefore the GdnHCl curing data alone is insufficient to provide an accurate estimate of *n*_0_. Here we present studies that allow us to accurately model the process of GdnHCl-mediated loss of the [*PSI* ^+^] prion using experimental techniques to measure the key parameters of asymmetric growth and propagon distribution. By so doing we have devised a robust stochastic model for estimating propagon numbers in yeast.

## Results

### A Stochastic Model for Accurately Estimating the Number of Propagons (Prion Seeds) in a [*PSI* ^+^] Yeast Cell

The earlier models we used to estimate *n*_0_, the number of propagons (prion seeds) in a [*PSI* ^+^] cell [19], [21] and referred to here as models A and B respectively, treated mother and daughter yeast cells as equivalent and assumed that propagons segregate with equal probability between the mother and daughter cell at cell division. However, *S. cerevisiae* cells divide asymmetrically and consequently the daughter cell that buds off from the mother cell, would receive proportionally fewer propagons. The most recent version of the model (model C) allows for both asymmetric cell division and unequal propagon segregation [22] although in none of the models were experimentally determined values for growth parameters used in the simulations.

In model C the probability that a propagon is passed to a daughter cell is *π* and thus the probability that a propagon is retained by the mother cell is (1−*π*). The probability that a cell contains prions at time *t* is then given as follows:
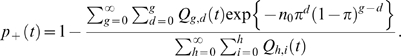



In order to evaluate p_+_(*t*) we consider every cell as having a history in which out of *g* past cell divisions (or generations), *d* were as daughter cells and (*g*−*d*) were as mother cells. *Q_g,d_*(*t*) is the expected number of cells that are at generation *g* with *d* daughter cell divisions at time *t*, and depends on cell lifetime distributions. These are allowed to differ between mother and daughter cells with the latter requiring a ‘maturation’ time. Detailed expressions for *Q_g,d_*(*t*) are given in Cole *et al.* [22] and **Table 1** summarises the parameters used in the model.

**Table 1:** The parameters used in curing model C and their use.

Parameter	Type	Description of use
***n*** **_0_**	Curing	Average number of propagons in a [*PSI* ^+^] cell at t = 0
***π***	Curing	Probability a propagon is transmitted to a daughter cell. Allows for unequal propagon distribution*.
**λ_M_**	Cell reproduction	Average time a mother cell takes to divide
**λ_D_**	Cell reproduction	Average time a daughter cell takes to divide
**β**	Cell reproduction	Extra cell division parameter that accounts for the variability in the time cells take to divide

* Value fixed at *π* = 0.5 in models A and B.

**Figure 1:**
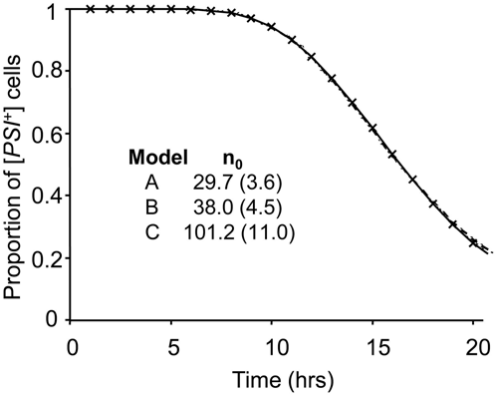
Simulation showing the proportion of [*PSI* ^+^] cells against time following the addition of 3 mM GdnHCl to a dividing yeast culture. The data (denoted by X) are simulated by assuming that cells start with an average of *n*_0_ = 100 propagons per cell. The time that a mother cell takes to divide follows a gamma distribution with a mean of 2 hr and standard deviation 0.28 hr. The total time that a daughter cell takes to divide also follows a gamma distribution, but with means 3 hr and standard deviation 0.35 hr. The probability that a propagon passes to a daughter cell is assumed to be 0.3. Three different models are shown fitted to the data: −·−·−· Model A [19]; - - - - Model B [21] and − Model C which gives an estimate of π = 0.3 (with standard error 0.02). The value of *n*_0_ estimated by each model is shown with standard errors in brackets.

To demonstrate that this new stochastic model is necessary, we used it to simulate data from a GdnHCl ‘curing’ experiment using the [*PSI* ^+^] strain YJW512 and then fitted each of the three models (A, B, C) to these data (**Figure 1**). Both model A and Model B fit the simulated data, but considerably underestimate *n*_0_, compared to model C [22]. In this simulation, Model C can be expected to give good estimates of the parameters because the data are simulated from it, but importantly is a more authentic representation of the biological processes that underpin the GdnHCl-induced loss of [*PSI* ^+^] from growing cells. Consequently its application to data generated from the growth of a [*PSI* ^+^] strain in GdnHCl should result in a more accurate estimate of *n*_0_. However, there is only enough information within the curing data to estimate two individual parameters reliably [22]. In our earlier study we estimated *n*_0_ and *π* from the curing data as these parameters were not experimentally established. In the simulation the remaining parameters (λ_M_, λ_D_ and β) were set to the values as determined experimentally below. In order to evaluate fully model C, we experimentally determined the key cell reproduction parameters.

### Cell Reproduction Parameters: Growth and Division of Individual [*PSI* ^+^] and [*psi* ^−^] Yeast Cells

**Figure 2:**
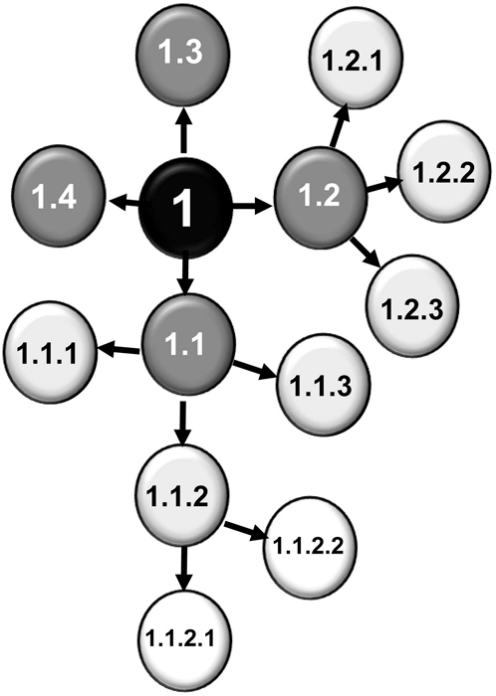
A ‘division tree’ diagram showing the growth and division of yeast cells from a single original mother cell. The original mother cell is labelled “1”. Each time a new bud (daughter) appeared on the original mother cell it was labelled 1.1, 1.2, 1.3 etc…and the time of appearance noted. When these daughter cells produced buds they were labelled 1.1.1, 1.1.2 etc… Two sets of data could be derived: a) the successive time taken for a cell to divide multiple times (mother cell reproduction times), and b) the time taken from emergence as a bud on a mother cell to subsequent first division (daughter cell reproduction times).

A quantitative assessment of the impact that both the presence of the [*PSI* ^+^] prion and/or 3 mM GdnHCl had on individual cell reproduction parameters was obtained using time-lapse microscopy. For [*PSI* ^+^] and [*psi* ^−^] cells in the presence and absence of 3 mM of GdnHCl, time-lapse microscopy was performed over 4–5 generations and the cell reproduction of individual mother and daughter cells of strain YJW512 was quantitatively assessed on the surface of solid YEPD medium. Further time-lapse microscopy examined cell reproduction when the prion [*PIN^+^*] was absent (i.e. in the [*pin* ^−^] strain YJW679). For each experiment a ‘division tree’ (**Figure 2**) was developed to track the emergence of daughter cells from individual mother cells and this allowed for an accurate estimation of the cell reproduction times of both the mother and daughter cells. The growth rates of the [*PSI* ^+^] strain YJW512 and its [*psi* ^−^] derivative in the absence or presence of 3 mM GdnHCl were determined (**Table 2**). The mother cell division time was defined as the time between successive new buds for an individual mother cell. As it was not possible to identify the precise timing of cytokinesis and cell separation coupled with the tendency of mother cells to reproduce again without a significant time lag, meant that the daughter cell division time was taken as the time between the appearance of a new bud on a mother cell and the time that bud first produced a daughter bud of its own.

**Table 2:** Cell reproduction data for the strains YJW512 [*PSI* ^+^] [*PIN* ^+^] and YJW679 [*PSI* ^+^] [*pin* ^−^] and their [*psi* ^−^] derivatives.

	Cell Type	Mean	SD	Size
**YJW512**				
**[** ***PSI*** **^+^]**	Mothers	1.23	0.25	82
	Daughters	1.46	0.33	57
**[** ***psi^−^*** **]**	Mothers	1.16	0.18	139
	Daughters	1.38	0.26	69
**Combined**	Mothers	1.19	0.21	221
	Daughters	1.42	0.29	126
**YJW512+GH**				
**[** ***PSI*** **^+^]**	Mothers	1.40	0.25	57
	Daughters	2.22	0.70	23
**[** ***psi*** **^−^]**	Mothers	1.42	0.25	54
	Daughters	2.22	0.69	20
**Combined**	Mothers	1.41	0.25	111
	Daughters	2.22	0.69	43
**YJW679+GH**				
**[** ***PSI*** **^+^]**	Mothers	1.27	0.19	57
	Daughters	1.90	0.49	20
**[** ***psi^−^*** **]**	Mothers	1.31	0.21	44
	Daughters	1.75	0.26	21
**Combined**	Mothers	1.28	0.20	101
	Daughters	1.82	0.43	41

Footnote: Shown is a summary of the statistics for the number of hours mother and daughter cells take to divide in rich growth medium (YEPD) without or with 3 mM guanidine hydrochloride (+GH). SD is the standard deviation, and size is the sample size.

The time that a mother cell takes to reproduce was assumed to have a gamma distribution with probability density function
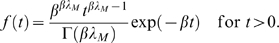



The additional time that a daughter cell takes to mature before starting to reproduce was taken to be independent of the subsequent time it takes to reproduce, and to have a gamma distribution with probability density function
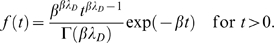



The mother cell lifetime distribution has mean *μ_M_* = *λ_M_* and variance *σ_M_* ^2^ = *λ_M_*/*β*. For daughter cells the corresponding mean and variance are *μ_D_* = *λ_M_*+*λ_D_* and *σ_D_* ^2^ = (*λ_M_*+*λ_D_*)*/β* (see **Table 1**).

Gamma cell lifetime distributions were fitted using maximum likelihood, to [*PSI* ^+^] and [*psi* ^−^] data sets separately and combined (**Table 3**). Using a likelihood ratio test, no significant difference in cell lifetime distributions for [*PSI* ^+^] and [*psi* ^−^] cells was observed. These data indicate that the [*PSI* ^+^] prion has no deleterious effect on cell lifetime distributions under these growth conditions. Consequently the [*PSI* ^+^] and the [*psi* ^−^] cell reproduction data were combined in order to provide a more accurate estimate of the key cell division parameters for our analysis.

**Table 3:** Parameter estimates from fitting the mother-daughter gamma distributions to the cell reproduction data for the YJW512 [*PSI* ^+^] and [*psi* ^−^] strains grown in YEPD.

Phenotype		*λ_M_*	*λ_D_*	*β*	*θ*
**[** ***PSI*** **^+^]**	Estimate (SE)	1.22 (0.026)	0.21 (0.043)	26.29 (3.509)	0.53 (0.009)
**[** ***psi*** **^−^]**	Estimate (SE)	1.16 (0.016)	0.21 (0.030)	31.03 (3.046)	0.55 (0.008)
**Combined**	Estimate (SE)	1.19 (0.015)	0.22 (0.026)	24.69 (21.880)	0.54 (0.007)

Footnote: SE is the standard error, for θ. This is a bootstrap standard error [23] with a bootstrap sample size of 10,000. The derivation of the growth rate or Malthusian parameter *θ*, is explained in the text.

The time-lapse data confirmed the findings originally made by Hartwell and Unger [24] that daughter cells take longer to divide than mother cells. The presence of 3 mM GdnHCl had a modest effect on cell reproduction, with cells reproducing more slowly in the presence of GdnHCl. For the strain YJW512, daughter cells took on average an additional 0.23 hr longer to reproduce in the absence of GdnHCl while in the presence of 3 mM GdnHCl, daughter cells took an extra 0.74 hr to reproduce. The growth rate *per se* does not influence the rate of dilution of propagon numbers [19]. Rather, the time dimension in the model is measured in generations and so is independent of growth rate. The cell division parameter estimates incorporated into the new stochastic model were those generated from mother and daughter cells grown in the presence of 3 mM GdnHCl (**Table 2**).

### Estimation of *n*_0_

**Figure 3:**
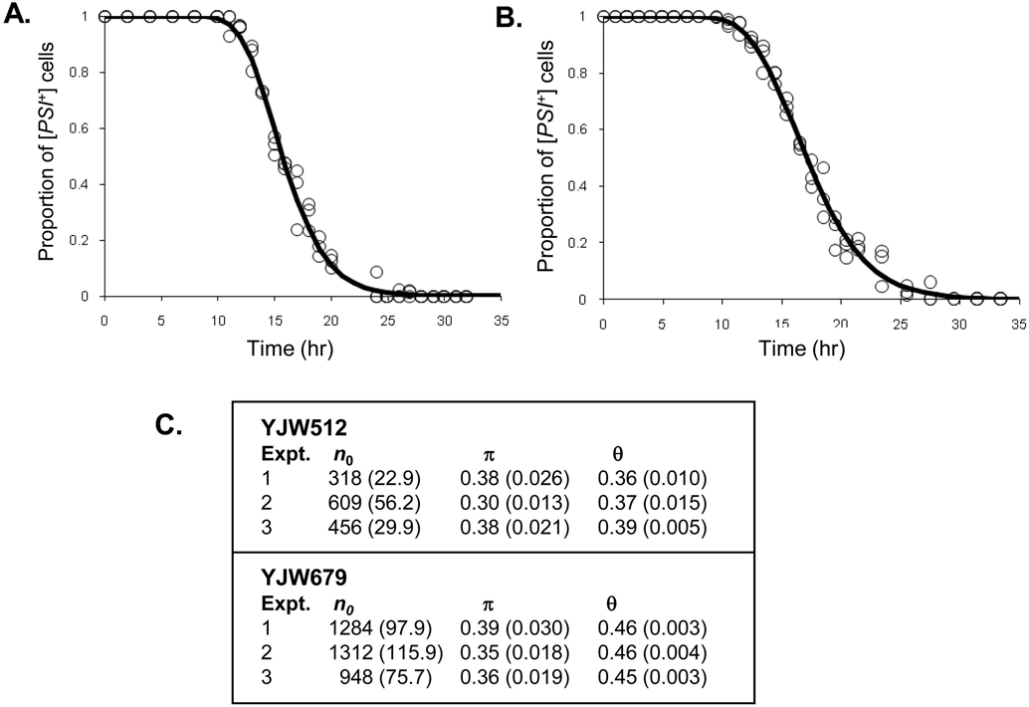
Observed proportion of [*PSI* ^+^] cells and fitted curve of p_+_(*t*). One set of data for each of the [*PSI* ^+^] strains (A) YJW512 and (B) YJW679 is shown. The curing parameter estimates for three independent experiments (1, 2, and 3) with 3 mM of GdnHCl are given in the table (C) with the values in brackets being the estimated standard errors for each parameter. Panels A and B represent one experiment while two other sets of data independently generated for these two strains are provided in Figure S1.

To evaluate the application of model C to the estimation of *n*_0_ in different [*PSI* ^+^] strains, three independent GdnHCl curing experiments were carried out on each of the two different [*PSI* ^+^] strains YJW512 and YJW679. The YJW679 strain lacked the [*PIN* ^+^] prion i.e. was [*pin^−^*]. For each experiment 3 mM GdnHCl was added at *t* = 0 to a growing culture and the proportion of cells in the culture that gave rise to [*PSI* ^+^] colonies was determined at the different subsequent time points up to *t* = 32 hr (**Figure 3**). The total number of viable cells at the various time points taken was also estimated. From this information the population growth rate or Malthusian parameter (*θ*) [23] was estimated as the slope of the linear regression of the logarithm of the estimated total number of cells on time. The value of *θ* was then used in conjunction with some of the information from the parameter estimates given in **Tables 3** **and** **4**. A near approximation of the exact relationship between the cell lifetime distributions and *θ* is given by:
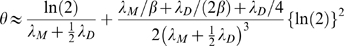

[23]. The estimates of *θ* in **Table 3** and **4** result from this expression.

**Table 4:** Parameter estimates from fitting the mother-daughter gamma distributions to the cell reproduction data for YJW512 [*PSI* ^+^] [*PIN* ^+^] and YJW679 [*PSI* ^+^] [*pin* ^−^] and their [*psi* ^−^] derivatives grown in YEPD+3 mM guanidine hydrochloride.

Phenotype		*λ_M_*	*λ_D_*	*β*	*θ*	*q*
**YJW512**						
**[** ***PSI*** **^+^]**	Estimate (SE)	1.42 (0.045)	0.75 (0.097)	12.04 (1.917)	0.40 (0.013)	
**[** ***pin*** **^−^]**	Estimate (SE)	1.44 (0.045)	0.73 (0.101)	13.04 (2.158)	0.40 (0.013)	
**Combined**	Estimate (SE)	1.43 (0.032)	0.74 (0.069)	12.50 (1.399)	0.40 (0.010)	0.52
**YJW679**						
**[** ***PSI*** **^+^]**	Estimate (SE)	1.28 (0.032)	0.60 (0.074)	21.33 (3.438)	0.45 (0.012)	
**[** ***pin*** **^−^]**	Estimate (SE)	1.31 (0.003)	0.42 (0.007)	23.25 (0.132)	0.46 (0.018)	
**Combined**	Estimate (SE)	1.29 (0.022)	0.51 (0.021)	21.68 (2.678)	0.46 (0.015)	0.40

Footnote: Shown are parameter estimates from fitting the mother-daughter gamma distributions to the data. SE is the standard error, for θ this is a bootstrap standard error [23], with bootstrap sample size 10,000. The derivation of the growth rate or Malthusian parameter,*θ*, is explained in the text. *q* = *λ_D_*/*λ_M_*, and is only given for values used in conjunction with curing experiments, as described in the text.

A combination of the time-lapse data and total cell counts obtained from the curing experiments was used to give estimates of cell reproduction thus enabling us to obtain estimates of the parameters *n*_0_ and *π* from the curing data. A combined likelihood analysis was considered, but because there is correlation between the total cell counts and the curing data, this results in biased estimates of *n*_0_ and *π*. An alternative strategy would be to estimate cell reproduction parameters solely from the time-lapse data, but this could introduce bias because cell growth in the curing experiments was in liquid medium while the time-lapse experiments were carried out with cells growing on the surface of an agar plate. However, there is reasonable agreement between the estimates of *θ* obtained in the two different ways (**Table 3**; **Figure 3C**). Although the estimates of *θ* are similar for the strain YJW512, a difference in *θ* values would have an effect on the estimates of *n*_0_ and *π*.

Consequently, the approach taken by Cole *et al.* [22] was used since this results in near unbiased estimates of *n*_0_ and *π* as shown through simulation studies. Let *q* = *λ_M_*/*λ_D_*. The estimates of *q* and *β* were derived from the time-lapse data (**Table 2**), and the estimate of *θ* from the total cell counts. The above approximation for *θ* can then be solved to estimate *λ_M_*. This approach is preferred to simply using the estimates obtained from the cell reproduction data (**Table 2**) alone. Variation in estimates of *q* and *β* were relatively small and so no account needed to be taken of this. Estimates of the cell reproduction parameters (*λ_M_*, *λ_D_* and *β*) were then used to fit model C. The data and fitted curves are shown in **Figure 3A, B** (see also **Figure S1**).

The estimates for *n*_0_ using model C range from 318 to 609 for YJW512 and range from 948 to 1312 for YJW679 (**Figure 3C**). These estimates are higher than those obtained with models A and B (see **Table S1**) because Model C accounts for asymmetric cell division and allows for unequal prion distribution. The estimates of *n*_0_ for the two strains were very different even though the only difference between the two strains was the presence or absence of the [*PIN* ^+^] prion. The [*PIN* ^+^] prion is essential for the *de novo* formation of other yeast prions but not for their continued propagation [25], [26]. It remains to be verified if the presence or absence of the [*PIN* ^+^] prion significantly impacts on propagon number *in vivo*.

We considered the possibility that exposing growing cells to 3 mM GdnHCl may have induced cell death and that this in turn might have impacted on the estimate of *n*_0_. Cell death has no effect on curing [22], but will effect both growth rate [20], [27] and *n*_0_ via *θ*. Consequently, the number of live cells at each time point taken was estimated by staining with phloxin B [28]. For either strain, the percentage of live cells was always ≥97% indicating that 3 mM GdnHCl did not adversely affect cell viability of either strain under the conditions used.

**Figure 4:**
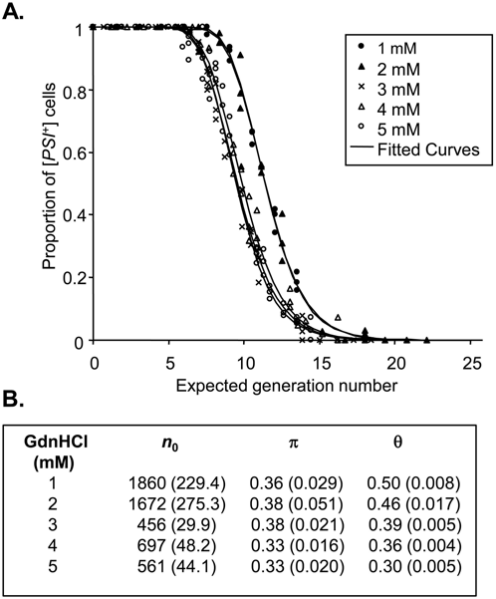
The effects of different concentrations of GdnHCl on the kinetics of elimination of the [*PSI* ^+^] prion from the strain YJW512. (A) The proportion of cells in the population at a given time is plotted against the expected generation number [27]. For each data set a fitted curve has been added. (B) Curing parameter estimates for different concentrations of GdnHCl. Values in brackets are the estimated standard errors for the parameters.

In the experiments described above, we made the assumption that the generation of new propagons was completely inhibited by 3 mM GdnHCl. However, this concentration of GdnHCl may cause elimination of [*PSI* ^+^] during curing without full inhibition of new propagon generation and this would lead to an over estimation of *n*_0_. If such blockage was incomplete in the presence of 3 mM GdnHCl, higher concentrations of GdnHCl would be expected to cure the cells of [*PSI* ^+^] faster, per generation. The experiments with YJW512 were therefore repeated using GdnHCl concentrations in the range 1 to 5 mM and the curing parameters estimated (**Figure 4**). Because the concentration of GdnHCl changes the growth rate, *θ*, of this strain (**Figure 4B**), the data were plotted against the expected generation number. Here we use an approximation to expected generation number that allows for variability in the time that cells take to divide and also allows for asymmetric cell division [27]. The approximation is given by:
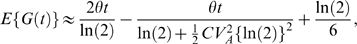
where 
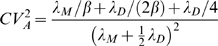
 [27].

In 1 mM and 2 mM GdnHCl, [*PSI* ^+^] was eliminated from cells more slowly in terms of expected generation number than for ≥3 mM while the curing curves for 3, 4 and 5 mM GdnHCl were essentially identical. These data therefore suggest that inhibition of propagon generation mediated by Hsp104 is complete at ≥3 mM GdnHCl in this [*PSI* ^+^] strain in a rich glucose-based medium such as YPD. Estimates of π also remained essentially constant at ≥3 mM GdnHCl (**Figure 4**). Hence at concentrations of GdnHCl<3 mM, one will get an over estimation of *n*_0_ because new propagon generation is not fully inhibited and therefore it takes longer for [*psi* ^−^] cells emerge thus leading to larger estimates of *n*_0_.

### [*PSI* ^+^] Propagons are Randomly Distributed at Cell Division

For model C, the probability of passing a propagon to a daughter cell (π) was estimated to be between 0.30–0.38 for strain YJW512 and between 0.37–0.44 for strain YJW679 (**Figure 3C**; **Figure 4**). In order to obtain a direct experimental determination of π, the proportion of propagons that are distributed to the daughter cell following budding, we used the method of Cox *et al.* [6], [22] to estimate *n*_0_.

**Figure 5:**
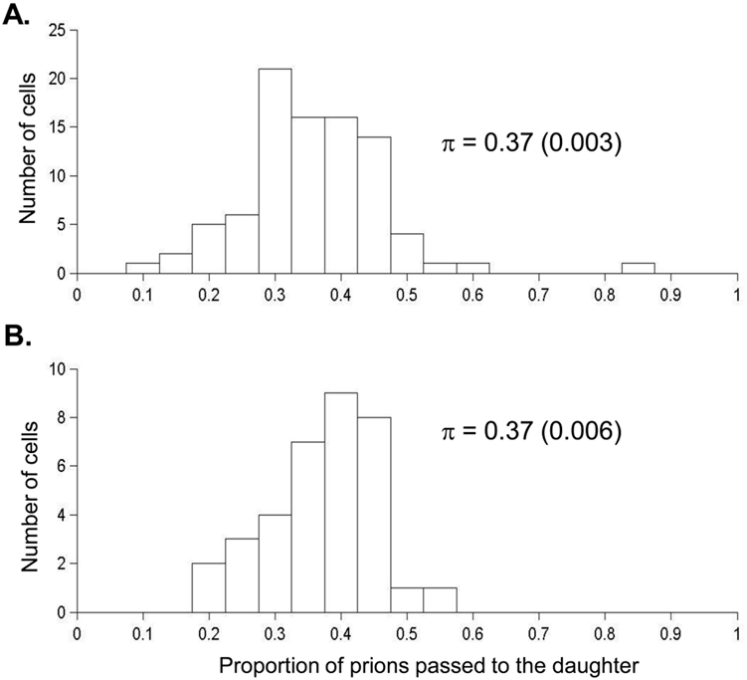
Proportion of prions passed on to the daughter cell for [*PSI* ^+^] strains YJW512 (A) and YJW679 (B). The maximum likelihood estimate of π (with standard error in brackets) shown is based on the assumption the number of prions passed on to a daughter cell follows a binomial distribution, as used in Model C (see [22].

Individual unbudded cells of two different [*PSI* ^+^] strains, YJW512 and YJW679 were micromanipulated onto the surface of a YEPD+3 mM GdnHCl agar plate and allowed to divide. The daughter cell was then micromanipulated away from the mother cell and both cells then allowed to grow into separate colonies. After 48 hr the whole colonies were resuspended and plated onto a defined medium to select for Ade^+^ cells. In theory, in the absence of cell death, the number of [*PSI* ^+^] colonies that arise will be equal to the total number of propagons in the original mother and daughter cell since the presence of GdnHCl does not lead to destruction of the [*PSI* ^+^] propagons [6], [22]. The resulting estimate of the total number of propagons for the mother and daughter pair was then used to estimate π. For both YJW512 and YJW679 this experiment gave a maximum likelihood estimate of for π of 0.37 (with standard errors of 0.003 and 0.006 respectively) i.e. the mother cell on average retains approximately 63% of the propagons and passes the remaining 37% to the daughter cell (**Figure 5**). This value is in very close agreement with the estimates for π from model C (**Figure 3**, **4**). Furthermore, that the experimentally-determined value of π was the same for both the [*PIN* ^+^] (YJW512) and [*pin* ^−^] (YJW679) strains shows that the differences observed in the estimate of *n*_0_ for these two strains does not reflect differences in propagon transmission from mother to daughter.

Estimates for *n*_0_ can also be obtained using this method by taking the average of the number of [*PSI* ^+^] colonies for the mother and daughter cell pair combined. The estimates of *n*_0_ were 248 (with standard deviation 98.9) for YJW512 and 384 (with standard deviation 119.6) for YJW679. These estimates are very variable and are much lower than those obtained from the data shown in **Figure 3**. This is because the method we used for estimating *n*_0_ [6], [22], [29] can be biased because cells were only allowed to develop into colonies for 24 hr before replating. The assumption made is that the resulting number of [*PSI* ^+^] cells in that colony should equal *n*_0_ i.e. the number of propagons present in the initial cell [6], [22], [29]. However, this means of estimating *n*_0_ would lead to an underestimation if there is an insufficient period of growth to ensure that there is only one propagon per cell. Furthermore, if the length of time the cells were left before plating onto solid growth medium was increased, cell death would potentially impact on the estimate of *n*_0_, as any propagons in a cell that dies are lost to that cell and reduce the estimated value of *n*_0_ concomitantly as [*PSI* ^+^] and other yeast prions cannot be transmitted to live cells following cell death.

The potential for such an underestimation of *n*_0_ was demonstrated by the following simulation: if *n*_0_ = 500, π = 0.37 and using the cell reproduction parameters as estimated for the strain YJW512 (**Table 2**), then it would take approximately 34 hr before there are 500 [*PSI* ^+^] cells. However, if only 3% of cells die and thus remove their associated propagons from the population, around 200 propagons are lost by 34 hr and this would lead to a substantial underestimate of *n*_0_. Thus, while cell death has little effect on the estimation of *n*_0_ when examining large populations of cells (as in **Figure 3**), it can have a significant effect when starting with a single cell as in the method of Cox [6], [22], [29]. Consequently this method should be used with caution if the objective is to obtain meaningful quantitative data on propagon number. Nevertheless it can be used for estimating π, as we have shown through simulation studies that there is very little bias in estimating π, due to the underestimation affecting both mother and daughter cell numbers equally.

To determine whether the observed relative distribution of propagons between mother and daughter cell reflected the cell volumetric differences between them at the time of cytokinesis, we estimated the respective relative cell volumes for mothers and daughters in both strains. The mean ratio of daughter to mother cell volumes was 0.40 (with standard deviation 0.033, but with minimum value 0.32 and maximum value 0.47). This value is reasonably close to our estimates of π, and is consistent with propagon transmission being based exclusively on random cytoplasmic transfer rather than requiring an active mechanism of transport from mother to daughter.

## Discussion

The continued propagation of the prion state of a protein in growing yeast cells requires the ability to generate and transmit molecular seeds – propagons - to new daughter cells. This must be done efficiently in order to match a cell doubling time of typically 1.5 to 2.0 hr. Failure to do so would soon see the pool of propagons depleted from a population and the emergence of prion-free cells. Although we are beginning to understand how propagon generation is achieved through the molecular chaperone and protein disaggregation activities of Hsp104 [30]–[32], the molecular composition of the ‘infectious’ propagon remains elusive. Studies with the mammalian prion PrP have indicated that non-fibrillar particles, which correspond to oligomers of between 14 and 28 PrP molecules, are the most efficient initiators of prion diseases and may therefore constitute the mammalian propagon [9]. No such information is yet available for any fungal prion.

Taking advantage of the fact that low concentrations of GdnHCl block the generation of new [*PSI* ^+^] propagons in growing cells through its inhibition of the chaperone activity of Hsp104 [11]–[13], [19], we have developed a modelling approach to estimating *n*_0_, the numbers of [*PSI* ^+^] propagons in the cell [19], [21], [22]. Our earlier modelling of the GdnHCl-mediated elimination of the [*PSI* ^+^] prion substantially underestimated this number due to the over-simplification of the basic growth characteristics of yeast cell reproduction. Of particular significance was the assumption that both cell division and propagon distribution between mother and daughter cells are symmetric. As we show here, this is not so in either case, even when cells are grown in the presence of GdnHCl. In this new study we have incorporated experimental data on asymmetric cell division together with measurement of the proportion of propagons that are transmitted to a daughter cell (π) to provide a more reliable and accurate estimate of *n*_0._

We also investigated whether the [*PSI*] status of a cell impacted on its reproduction and found that the presence of [*PSI* ^+^] prions has no deleterious effect on cell reproduction, at least under our conditions. This finding suggests that the significant reduction in available soluble Sup35p for translation termination typically seen in strong [*PSI* ^+^] variants does not impair growth and cell division. That there are approximately 5 molecules of Sup35p for every one molecule of its translation termination partner, Sup45p (T. von der Haar, personal communication) might explain this discrepancy i.e. no more than 20% of Sup35p in the cell is engaged in termination. In addition, one (or more) of the high mol. wt. forms of Sup35p found in a [*PSI^+^*] cell may still be functional in termination.

Others have argued that in strains of *S. cerevisiae* isolated from their natural habitat, both the [*PSI* ^+^] and [*URE3*] prions may have had a net deleterious effect on cell reproduction which, albeit subtle, leads to a failure to find wild strains containing these prions [33], [34]. This may not be the case for the [*PIN^+^*] prion however since a number of wild strains of *S. cerevisiae* that are [*PIN^+^*] have been found [33], [34]. Our detailed analysis of cell reproduction parameters failed to reveal any negative impact of [*PSI* ^+^] on cell growth and division, but it should be acknowledged that these experiments are carried out under nutrient-rich conditions rarely encountered by a yeast cell in the wild.

### Distribution of Propagons During Cell Division

We established experimentally the relative proportion of [*PSI^+^*] propagons that are transmitted from a mother to a daughter cell on cell division. This was done under conditions where the cells are still able to divide but are unable to generate new propagons because they were grown in the presence of 3 mM GdnHCl, a concentration that completely inhibits new propagon generation (**Figure 4**). This experiment showed that the mother retains about two thirds of the propagons whilst passing the remaining one third to the daughter cell. From our estimates for *n*_0_, this would suggest that the daughter cell normally inherits more than sufficient numbers of propagons (*n*_0_>100) to ensure on-going prion propagation, especially given that propagon numbers double approximately every 20 min when a cell is released from a GdnHCl-mediated block in propagon generation [35].

The relative distribution of propagons between mother and daughter cells was similar to the relative approximate volumes of mother and daughter cells at the point of cell division, with the mother cell being on average 1.5 times the volume of the daughter. This suggests that there is no active transport of propagons into the daughter cell during cell division, but rather that there is a passive transfer via distribution of cytoplasm between the dividing cells. There has been no evidence to date of yeast prion proteins being physically associated with any cellular structures, although there are several reports implicating components of the cytoskeleton in the process of [*PSI* ^+^] prion generation and propagation in yeast [36]–[38].

### Accurate Estimation of *n*_0_

The work we report here constitutes a considerable advance on that of Cole *et al.* [22], which established model C, but which only considered rudimentary model fitting to historical data. We have also incorporated the advances reported by Ridout *et al.* [23] and Cole *et al.* [27] who respectively developed better approximations for the growth rate and generation number of yeast cells that allow for variation in the times cells take to divide, and asymmetric cell division.

Incorporating asymmetric cell division and unequal propagon distribution into our stochastic model and fitting the separate GdnHCl-induced [*PSI^+^*] elimination data for the strain YJW512 ([*PSI* ^+^/*PIN* ^+^]) resulted in estimates of *n*_0_ varying from 318 to 609, much greater numbers than we estimated using the earlier models A [19] and B [21]. The relatively large variance in the estimates of *n*_0_ for a given [*PSI^+^*] strain that we observe may reflect the differences in the cells selected for examination. For each independent experiment we carried out with a given [*PSI^+^*] strain, the cultures were derived from different colonies although for any given population one would expect to see similar variation between cells.

Three identical experiments performed using a second [*PSI^+^*] strain YJW679 gave much higher values of *n*_0_ (905 to 1346), indicating a consistently greater number of propagons than its close relative YJW512. YJW679 has an identical genotype to YJW512, only differing in its [*PIN^+^*] prion status: YJW679 is [*pin* ^−^]. While it remains to be established whether the presence or absence of the [*PIN* ^+^] prion directly affects the number of [*PSI* ^+^] propagons in other strain pairs, we show here that these differences are not due to differences in the efficiency of propagon transmission to daughter cells. One plausible explanation for the increased numbers of [*PSI* ^+^] propagons observed in the [*pin* ^−^] strain is that a significant fraction of the Hsp104 chaperone that would normally be engaged in disaggregating the Rnq1p aggregates present in the [*PIN* ^+^] strain, is available in a [*pin* ^−^] strain to facilitate the more efficient breakdown of Sup35p prion aggregates. This might in turn would generate a higher number of Sup35p fragments i.e. propagons.

### Implications for Prion Propagation

In the future, modelling strategies will be able to use data from experiments such as those we have reported here to help explain how it is possible to go from native protein to high molecular weight aggregate via an infectious propagon in a dynamic system. But the propagon, as a physical entity, remains elusive for all three well established native yeast prions [*PSI^+^*] [*PIN^+^*] and [*URE3*]. That yeast prions require Hsp104 to propagate [14]–[16] means that the approach we have taken with [*PSI^+^*] to estimate the number of propagons can also be applied to the other native prions. The only limitation is the relative difficulty in scoring the [*PIN^+^*] prion-associated phenotype in order to generate sufficient numbers to make the model meaningful.

Our study has also demonstrated the importance of including unequal cell division when modelling the curing of [*PSI^+^*] cells by the introduction of the parameter π. It has been assumed that π is constant, but this may not necessarily be so. GdnHCl does not block the aggregation of Sup35p in a [*PSI^+^*] cell [35] and consequently such polymers may increase in size during the course of a GdnHCl curing experiment. If this is the case, then the Sup35p polymers may then become too large to transfer efficiently to a daughter cell. A consequence would be that π would decrease over time and using model C which assumes constant π, this would underestimate *n*_0_. Although we have no direct evidence for such a decrease in π in our standard GdnHCl curing experiments, in an earlier study we showed that applying α-factor to [*PSI^+^*] cells resulted in an apparent reduction in π [20]. This effect could be due to the unusually shaped daughter cells (‘schmoos’) or due to the propagons growing abnormally large in the 12 hr during which growth of the cells are arrested by α-factor.

Our revised higher value for *n*_0_ also has important implications for the better understanding of the molecular events that lead to the spontaneous appearance of prion-free cells in a growing population. Although no accurate estimate for the frequency with which [*PSI^+^*] is lost has yet been reported, it is certainly lower than 2×10^−4^ [39] and is probably nearer the frequency for spontaneous nuclear gene mutations i.e. ∼10^−6^ [40]. If the probability of generating a [*psi* ^−^] cell is 10^−6^ then, with the value of π we estimate here of 0.37 and using our stochastic model C, this would give an *n*_0_ of around 30. For an *n*_0_ value of 100 and π = 0.37 then the probability of generating a [*psi* ^−^] cell drops to around 10^−20^. Consequently the loss of [*PSI^+^*] from a cell can not be due to a random failure to transmit at least one propagon to the daughter cell during cell division, but rather other molecular or cellular events must trigger the loss, the nature of which can now be established.

## Materials and Methods

### Yeast Strains

The yeast strains used in this study were as follows:

YJW512: *MATa leu2-3,-112*, *ura3-1*, *his3-11,-15*, *trp1-1*, *can1-100*, *ade1-14*
YJW679: *MATa leu2-3,-112*, *ura3-1*, *his3-11,-15*, *trp1-1*, *can1-100*, *ade1-14*

For YJW512 both [*PSI* ^+^] [*PIN^+^*] and [*psi* ^−^] [*PIN^+^*] derivatives were used, while for strain YJW679 both [*PSI^+^*] [*pin* ^−^] and [*psi* ^−^] [*pin^−^*] derivatives were used.

### Growth Medium

Yeast strains were grown at 30°C with shaking (200 rpm) in YEPD, a rich liquid medium (1% (w/v) yeast extract, 1% (w/v) bacto-peptone, 2% (w/v) glucose) with or without GdnHCl. Cells were plated onto either YEPD solid medium (as above including 2% (w/v) agar), ¼ YEPD solid medium (0.25% (w/v) yeast extract, 1% (w/v) bactopeptone, 2% (w/v) glucose, 2% (w/v) agar) or adenine deficient synthetic complete medium supplemented with 5% (v/v) liquid YEPD (2% (w/v) glucose, 0.67% (w/v) yeast nitrogen base with ammonium sulphate, 0.2% (w/v) adenine drop-out mixture [Formedium, UK], 5% (v/v) YEPD liquid medium, 2% (w/v) agar).

### Measuring Growth and Reproduction of Individual Yeast Cells

Autoclaved glass slides were placed in sterile Petri dishes and covered with 10 ml of molten YEPD solid medium (with or without GdnHCl) to create a thin layer of agar on the slide surface. The yeast strain under test was inoculated into 50 ml fresh liquid YEPD and grown at 30°C with shaking (200 rpm) until the culture reached mid-exponential phase. 100 µl of this culture was plated onto the Petri dish containing the embedded slide and allowed to dry for 30 min. The slide was removed from the Petri dish, a cover slip placed on top and the excess solid medium trimmed from around the edges of the cover slip. The cover slip was then sealed using molten VALAP (1∶1∶1 mixture of vaseline, lanolin and paraffin wax) to provide a semi-gas permeable seal. The individual yeast cells were observed under an oil immersion 63× objective lens in a 30° incubation chamber mounted on a Leica Multi-Dimension Workstation (MDW) microscope attached to a digital camera. Images of the growing yeast cells were recorded every 180 sec for 12 hr. Cells were kept in focus by recording additional images in 1 µm stages above the Z-axis. Manipulation of the resulting images was performed using the computer program Image J (http://rsb.info.nih.gov/ij/index.html) which stacked the individual images to allow scrolling so that the time (to the nearest 180 sec) of the emergence of buds from mother cells could be recorded.

### Measuring Cell Volume of Individual Yeast Cells

Using brightfield images taken from the time-lapse microscopy experiments, the length and width of 50 [*PSI* ^+^] and 50 [*psi* ^−^] mother and daughter pairs were measured post cytokinesis using Image J. Using these measurements the approximate volume of each cell was calculated by assuming them to be cylinders with all cells having the same height and elliptic cross-sections. The ratio of the volume of daughter cells to volume of mother cells was then estimated as (w_D_×l_D_)/(w_M_×l_M_), where w_D_ is the width of the daughter cell, l_D_ is the length of the daughter cell, w_M_ is the width of the mother cell and l_M_ is the length of the mother cell.

### Propagon Distribution Between Mother and Daughter [*PSI* ^+^] Cells

The proportion of propagons passed from mother to daughter cells during cell division was determined using the method described in [6]. The test [*PSI* ^+^] strain was grown in YEPD at 30°C to exponential phase and the cells plated onto solid YEPD medium containing 3 mM GdnHCl. Using a Mark III Singer Micromanipulator, individual yeast cells were separated into rows on the solid medium and incubated at 30°C for 2–3 hr to allow a single round of cell division. Micromanipulation was then used to separate the resulting daughter from the mother cell, before returning the cells to 30°C for a further 24 hr incubation. The small colonies that developed were removed in their entirety using shortened micropipette tips and re-suspended in 200 µl of sterile PBS pH 7.4 by vigorous vortexing. The resulting cell suspension was plated onto solid adenine-deficient synthetic medium supplemented with 5% (v/v) YEPD medium (S.C. −Ade+5% YEPD) and incubated at 30°C for 3–5 days. The number of resulting white Ade^+^ colonies counted was a direct representation of the number of propagons present in the cell. That Ade^+^ colonies were [*PSI* ^+^] was confirmed by plating onto ¼ YEPD+3 mM GdnHCl medium [6].

### Curing the [*PSI* ^+^] Prion by GdnHCl

To study the elimination of the [*PSI^+^*] prion from a given strain, 100 µl of culture growing exponentially in YEPD at 30°C was used to inoculate 50 ml of fresh YEPD liquid medium containing 1–5 mM GdnHCl and grown with shaking at 30°C. At regular intervals (up to *t* = 32 hr), three separate 100 µl samples were taken and diluted appropriately in sterile PBS pH 7.4, spread onto ¼ YEPD solid medium (typically 100–300 colony-forming units per plate) and incubated at 30°C for 3–5 days to determine the proportions of [*PSI^+^*] and [*psi^−^*] cells in the culture. This composition was evaluated from counts of white and red colonies based on a marker system that exploits the suppression of the *ade1-14* allele that was present in all yeast strains used in this study. Only wholly red colonies were scored as [*psi* ^−^] [19]) with red/white sectored colonies being scored as [*PSI* ^+^].

### Monitoring Cell Death

Cultures were grown to mid-exponential phase in YEPD medium containing 10 µmol phloxin B (Sigma; [28] to which 3 mM GdnHCl was added as required. Culture samples were taken every 2 hr and the numbers of live (transparent) and dead (stained red) cells counted microscopically using a haemocytometer and the percentage live cells scored.

## Supporting Information

Table S1 Estimates of n0 for Models A, B and C.
(0.03 MB DOC)

Figure S1 Observed proportion of [PSI+] cells and fitted curve of p+(t).
(0.15 MB DOC)
