# Supplementary material for: The Number and Transmission of [PSI +] Prion Seeds (Propagons) in the Yeast Saccharomyces cerevisiae
Source: PLoS One. 2009 Mar 5;4(3):e4670. doi: 10.1371/journal.pone.0004670 (PMC2650407; doi:10.1371/journal.pone.0004670)
Supplement: Table S1 — Estimates of n0 for Models A, B and C. (0.03 MB DOC) [file pone.0004670.s001.doc]

**Table S1: Estimates of *n*_0_ for Models A, B and C**

| **YJW512** |  |  |  |
| --- | --- | --- | --- |
| Exp | A | B | C |
| 1 | 123 (12.7) | 170 (19.7) | 318 (22.9) |
| 2 | 80 (7.9) | 111 (8.4) | 609 (56.2) |
| 3 | 138 (17.4) | 232 (25.2) | 456 (29.9) |
| **YJW679** |  |  |  |
| Exp | A | B | C |
| 1 | 508 (98.2) | 847.3 (140.1) | 1284 (97.9) |
| 2 | 316 (38.7) | 385 (48.8) | 1312 (115.9) |
| 3 | 279 (46.8) | 365 (53.3) | 948 (75.7) |

Shows the estimates of *n*_0_ (the average number of prions at the start of the experiment) using Model A [19], Model B [21] and Model C [22] for each of the three experiments shown in **Figure 3** and **Figure S1**.
