# Supplementary material for: The Number and Transmission of [PSI +] Prion Seeds (Propagons) in the Yeast Saccharomyces cerevisiae
Source: PLoS One. 2009 Mar 5;4(3):e4670. doi: 10.1371/journal.pone.0004670 (PMC2650407; doi:10.1371/journal.pone.0004670)

**Figure S1: Observed proportion of [*PSI*^+^] cells and fitted curve of p_+_(*t*).**

Essentially the same experiment as shown in **Figure 3**. Data from two additional, independent experiments are shown for both [*PSI*^+^] strains YJW512 and YJW679.


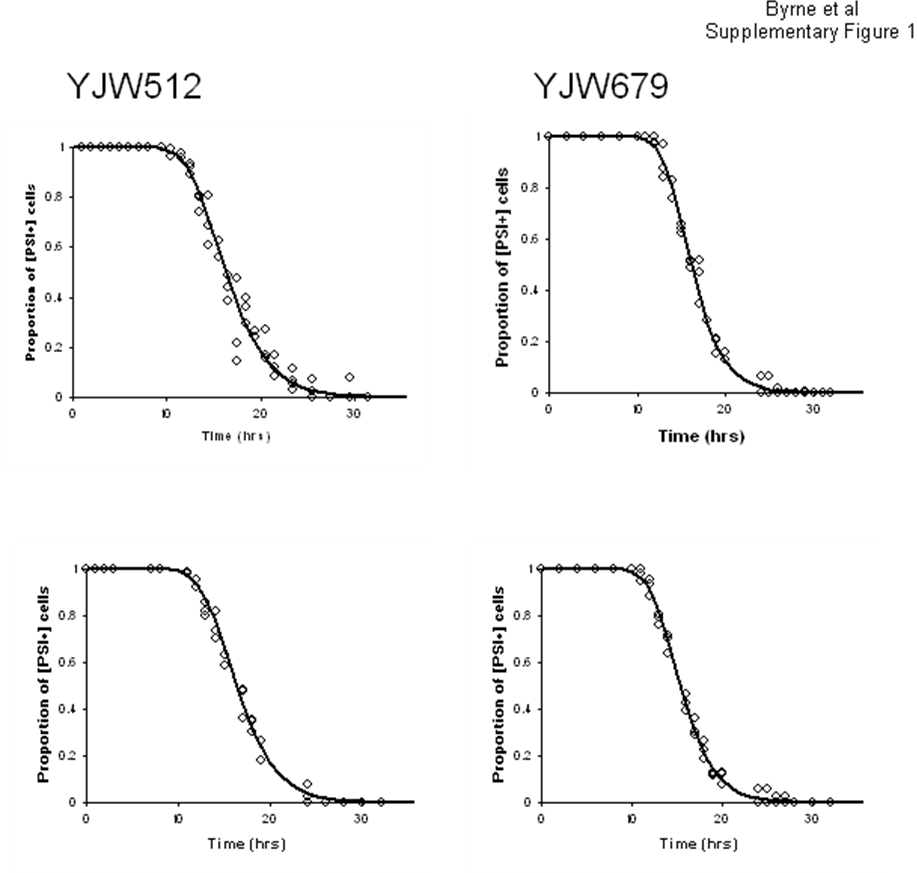

Supplement: Figure S1 — Observed proportion of [PSI+] cells and fitted curve of p+(t). (0.15 MB DOC) [file pone.0004670.s002.doc]
